# Fucoidan from Marine Brown Algae Inhibits Lipid Accumulation

**DOI:** 10.3390/md9081359

**Published:** 2011-08-10

**Authors:** Min-Kyoung Park, Uhee Jung, Changhyun Roh

**Affiliations:** Radiation Biotechnology Research Division, Advanced Radiation Technology Institute (ARTI), Korea Atomic Energy Research Institute (KAERI), 1266 Shinjeong-dong, Jeongeup-si, Jeollabuk-do 580-185, Korea; E-Mails: mkpark@kaeri.re.kr (M.-K.P.); uhjung@kaeri.re.kr (U.J.)

**Keywords:** marine brown algae, lipid inhibition, lipolysis, hormone sensitive lipase

## Abstract

In this study, we elucidated the inhibitory effect of fucoidan from marine brown algae on the lipid accumulation in differentiated 3T3-L1 adipocytes and its mechanism. The treatment of fucoidan in a dose-dependent manner was examined on lipid inhibition in 3T3-L1 cells by using Oil Red O staining. Fucoidan showed high lipid inhibition activity at 200 μg/mL concentration (*P* < 0.001). Lipolytic activity in adipocytes is highly dependent on hormone sensitive lipase (HSL), which is one of the most important targets of lipolytic regulation. Here, we examined the biological response of fucoidan on the protein level of lipolysis pathway. The expressed protein levels of total hormone sensitive lipase (HSL) and its activated form, phosphorylated-HSL were significantly increased at concentration of 200 μg/mL fucoidan. Furthermore, insulin-induced 2-deoxy-d-[^3^H] glucose uptake was decreased up to 51% in fucoidan-treated cells as compared to control. Since increase of HSL and p-HSL expression and decrease of glucose uptake into adipocytes are known to lead to stimulation of lipolysis, our results suggest that fucoidan reduces lipid accumulation by stimulating lipolysis. Therefore, these results suggest that fucoidan can be useful for the prevention or treatment of obesity due to its stimulatory lipolysis.

## 1. Introduction

Obesity is a chronic metabolic disorder caused by an imbalance between energy intake and expenditure. It has become a major obstacle of improving human health and life quality due to the known risk factors for many chronic diseases. It is characterized by hyperplasia and hypertrophy, and is regarded as a major factor for cardio-metabolic disturbances such as hypertension, atherosclerosis, type 2 diabetes and dyslipidemia [1–3]. In general, obesity is associated with the extent of adipocyte differentiation, intracellular lipid accumulation and lipolysis [4,5].

Marine brown algae have long made up a key part of the Asian diet and are also consumed in other parts of the world. It has often been used as a food for people who are sick and has been credited with health-giving properties. Today, marine brown algae supplements for human use are usually considered to be sources of therapeutic benefits [6]. Especially, Fucoidan is a sulfated fucose containing polysaccharide extracted from marine brown algae [7]. During the past years, fucoidan extracted from various species has been recognized to possess biological properties; anti-coagulant, anti-thrombotic, anti-tumor, anti-inflammatory and anti-virus activities [8]. The anti-coagulant activity of sulfated polysaccharide from *Fucus evanescens* was similar to that of heparin. Anti-coagulant properties of fucoidan are determined by thrombin inhibition mediated via plasma antithrombin III [9]. Fucoidan from *Mekabu*, a dietary alga, exerts anti-tumor activity possibly through enhancing the Th1 cell and NK cell responses [10]. Fucoidan, a sulfated polysaccharide isolated from an edible brown alga *Undaria pinnatifida*, was shown to be a potent inhibitor of the *in vitro* replication of herpes simplex virus type 1 (HSV-1) [11–13].

Recently, there has been increasing interest in potential biological activity against fat inhibition from fucoidan, which is an adipogenic reduction of fucoidan-declined lipid accumulation in adipocytes. Fucoidan can be used for inhibiting fat accumulation, which is mediated by decreasing aP2, ACC, and PPARγ gene expression [14]. Fucoidan treatment inhibits adipocyte differentiation, evidenced by decreased lipid accumulation and down regulation of adipocyte markers via mitogen-activated protein kinase (MAPK) signaling pathways. As of yet, however, no study has clearly investigated the inhibitory effects of fucoidan on lipid accumulation through the regulation of lipolysis. Lipolysis is a prerequisite for lipid accumulation via the regulation of hormone sensitive lipase (HSL) as a rate limiting enzyme, which is the hydrolysis of triglycerides into free fatty acids.

In this study, we examined whether fucoidan from *Fucus vesisulosus* inhibits lipid accumulation in 3T3-L1 adipocytes via the stimulation of lipolysis. First, we quantified the accumulation of lipid droplets in 3T3-L1 cells in a dose-dependent manner using Oil Red O staining. Second, we showed the stimulatory effect of fucoidan on the protein expression of HSL and its activated form, p-HSL by western blotting assay, which function as the key rate-limiting enzyme on lipolysis. Furthermore, we examined insulin-induced glucose uptake into 3T3-L1 adipocytes as a measure of lipolytic effect of fucoidan. Finally, we confirmed inhibitory effect of fucoidan on triglyceride accumulation in 3T3-L1 cells. As a result, we demonstrated that fucoidan may reduce lipid accumulation through the stimulation of lipolysis and may be a candidate for an anti-obesity agent.

## 2. Results and Discussion

### 2.1. Cell Viability and Lipid Inhibitory Effect of Fucoidan on 3T3-L1 Adipocyte Differentiation

**Figure 1.**
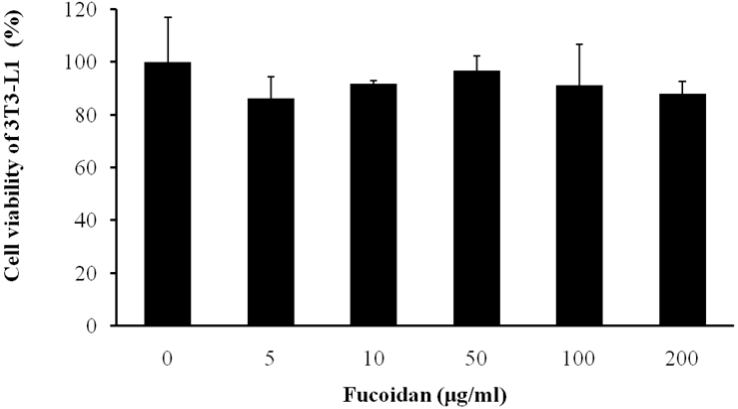
Effect of fucoidan on viability of 3T3-L1 adipocytes. Cells were treated with various concentration of fucoidan for 4 days. On day 8, cell viability was determined via CCK-8 assay. All values are the mean ± standard deviation (SD) from five independent experiments.

We first examined whether fucoidan has an influence on cell viability of 3T3 adipocytes using a CCK-8 assay. Fucoidan did not have toxicity at concentrations in a dose-dependent manner (Figure 1).

**Figure 2.**
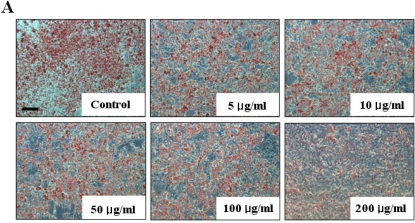

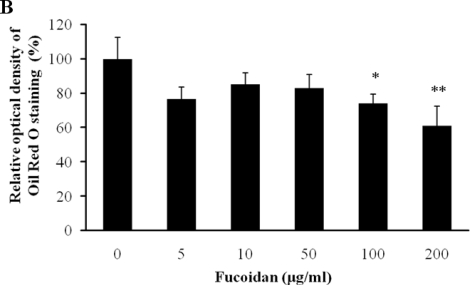
Effect of fucoidan on 3T3-L1 adipogenic differentiation. 3T3-L1 preadipocytes were treated with various concentration of fucoidan until Day 8, and change of adipocyte differentiation was determined with Oil Red O staining: (**A**) Microscopic observation of 3T3-L1 cells; and (**B**) Quantification analysis by Oil Red O staining. The eluted Oil Red O dye with isopropanol was quantified by measuring optical density at 500 nm with UV-spectrophotometer. Values given are the mean ± standard deviation of five independent experiments. Scale bar represents 100 μm (* *P* < 0.05, ** *P* < 0.001).

**Figure 3.**
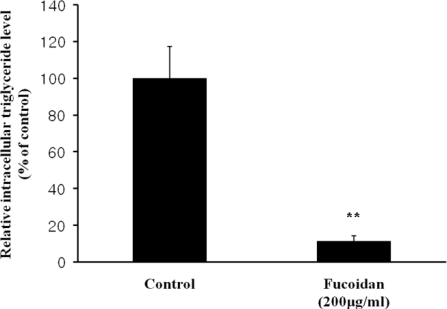
Triglyceride accumulation in 3T3-L1 adipocytes treated with fucoidan. The triglyceride contents in the lysates of 3T3-L1 cells were analyzed by using the TG-S reaction kit. Data represent the mean ± SD from three independent tests (** *P* < 0.001).

The inhibitory effect of fucoidan on differentiation of 3T3-L1 preadipocytes was then examined by Oil Red O staining. The microscopic inspection of fucoidan-treated adipocytes after Oil Red O staining revealed a significant reduction in the lipid droplets and accumulation of intracellular lipid (Figure 2A). The lipid accumulation was decreased by 16.5% (*P* < 0.05) at 100 μg/mL and 52.2% (*P* < 0.001) at 200 μg/mL of fucoidan, respectively (Figure 2B). Triglyceride assay was performed that fucoidan could inhibit triglyceride accumulation in 3T3-L1 adipocytes (Figure 3). Fucoidan exhibited 86% decrease in triglyceride accumulation compared to the control. Therefore, fucoidan showed anti-adipogenic properties as evidenced by decreased lipid and triglyceride accumulation in 3T3-L1 cells.

### 2.2. Expression Level of Lipolytic Gene

**Figure 4.**
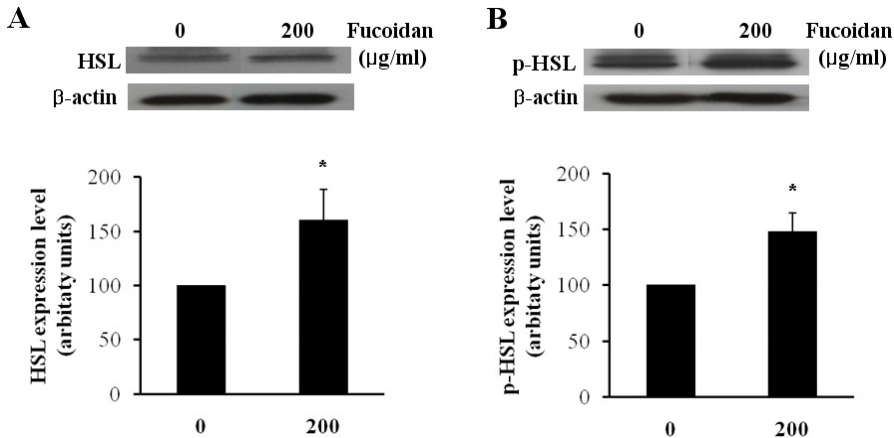
Effect of fucoidan on expressions of HSL and p-HSL. The protein expressions of (**A**) HSL and (**B**) p-HSL were examined by western blotting (top) and the expression levels were quantified by densitometry (bottom) (* *P* < 0.05).

Lipolytic activity in adipocytes is highly dependent on hormone sensitive lipase (HSL), which is one of the most important targets of lipolytic regulation. The subsequent phosphorylation and activation of HSL result in an increase of the hydrolysis of stored triacylglycerol into mono-acylglycerol and free fatty acids [15–17]. To evaluate the effect of fucoidan on the regulation of lipolysis, we investigated the protein level of HSL and phosphorylated HSL (p-HSL) by western blotting analysis. In fully differentiated 3T3-L1 adipocytes, fucoidan treatment at 200 μg/mL increased HSL and p-HSL level by approximately 1.47 and 1.59 times as compared to the controls (Figures 4A,B). Kim group reported that the treatment of gensonosides or EGCG prevented obesity via the up-regulated expression of HSL mRNA [18]. Cha group showed that methanol extract from Luffa cylindrical (LCM) stimulates the lipolysis through the induction of HSL resulting in the reduced lipid accumulation [19]. Therefore, our results suggest that fucoidan may induce lipolysis by increasing the protein levels of HSL and p-HSL.

### 2.3. Inhibitory Effect of Fucoidan on Insulin-Induced Glucose Uptake

**Figure 5.**
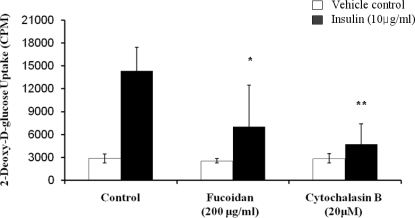
Inhibitory effect of fucoidan on glucose uptake into 3T3-L1 adipocytes. Differentiated 3T3-L1 cells were incubated with 10 μg/mL insulin in the presence or absence of 200 μg/mL fucoidan, and then assayed for 2-deoxy-d-[^3^H] glucose uptake. 20 μM Cytochalasin B was used as a positive control for inhibition of glucose uptake. The mean ± SD from at least three replicate experiments (* *P* < 0.05, ** *P* < 0.001).

The inhibitory activity of fucoidan on glucose uptake in 3T3-L1 adipocytes was assessed by using 2-deoxy-d-[^3^H] glucose uptake quantitative assay (Figure 5). In the absence of insulin, fucoidan had no significant effect. However, it demonstrated its inhibitory effect on glucose uptake. The glucose uptake was greatly enhanced in the presence of 10 μg/mL insulin. Insulin, which is a central hormone to regulate fat metabolism in the body, causes cells in adipocytes tissue to take up glucose from the blood, stimulating glucose utilization by its anti-lipolytic effect on adipose tissue [20]. However, the insulin-induced glucose uptake was decreased by fucoidan at 200 μg/mL (51% decreases) and cytochalasin B, which is a positive control for glucose uptake inhibition. Therefore, our results further present that fucoidan inhibits lipid accumulation by decreasing glucose uptake.

In summary, the findings of this study clearly demonstrated the inhibitory effect of fucoidan extracted from Fucus vesisulosus on lipid accumulation in 3T3-L1 adipocytes via stimulatory lipolysis. The stimulation of lipolysis was mediated by increasing the protein expression of HSL and p-HSL, which are essential regulation factors for lipolysis, and by decreasing glucose uptake into 3T3-L1 adipocytes. Therefore, we suggest that fucoidan is a promising candidate for an anti-obesity agent by blocking lipid accumulation and stimulating lipolysis.

## 3. Experimental Section

### 3.1. Materials

The preadipogenic cell line 3T3-L1 was obtained from the American Type Culture Collection (ATCC, CL-173). Dulbecco’s modified Eagle medium (DMEM), bovine calf serum (BCS), fetal bovine serum (FBS), phosphate-buffered saline (PBS) and trypsin–EDTA were acquired from Gibco (Gaithersburg, MD, USA). Penicillin–streptomycin, dexamethasone (DEX), 3-isobutyl-1-methylxanthine (IBMX), insulin and Oil Red O were purchased from Sigma (St, Louis, MO, USA). Fucoidan, extracted from focus vesiculosus, was provided by Sigma (St, Louis, MO, USA). CCK-8 was purchased from Daeil Lab Service, Ltd. (Seoul, Korea).

### 3.2. Cell Culture and Differentiation

3T3-L1 preadipocyte cells purchased from ATCC (Manassas, VA, USA) were cultured, maintained and stimulated to differentiate as described previously [16]. Cells were initially maintained in DMEM with 10% BCS, and 1% (v/v) penicillin-streptomycin (100 units/mL penicillin and 100 μg/mL streptomycin) at 37 °C in 5% CO_2_. At 2 days post-confluence, 3T3-L1 cell differentiation was induced by treating confluent cells with a hormone mixture containing 10% FBS, 10 μg/mL insulin, 0.5 mM IBMX and 0.5 μM DEX. To investigate the effects of fucoidan on fully differentiated adipocytes, fucoidan concentrations of 0, 5, 10, 50, 100 and 200 μg/mL were applied to the cells. Two days later, the medium was placed into DMEM supplemented with only 10 μg/mL insulin and 10% FBS. The same concentrations of fucoidan were supplemented at two-day intervals when the culture medium was replenished. After adipocyte differentiation, the cells were stained with Oil Red O staining, which is an indicator of cell lipid content. Cells were washed with phosphate-buffered saline, fixed with 10% buffered formalin and stained with Oil Red O solution (0.5 g in 100 mL isopropanol) for 1 h and observed under a microscope. For quantification, the staining solution was removed, the dye retained in the cells was eluted into isopropanol, and OD_500_ was determined using a spectrophotometer (UVIKON XS, SECOMAM, France).

### 3.3. Cell Viability

Cell viability was assessed using a CCK-8 assay. 3T3-L1 adipocyte cells were treated with fucoidan at 0, 5, 10, 50, 100, and 200 μg/mL for 4 days. After adipocyte differentiation, CCK-8 solution was added, and cells were incubated at 37 °C for 30 min. Finally, absorbance was measured at 450–650 nm to obtain the percentage of viable cells.

### 3.4. Western Blotting

Cultured differentiated cells were lysed in an ice-cold lysis buffer (1 M Tris, 5 M NaCl, 100% NP-40, 1 M NaF, 100 mM Na_2_NO_4_, 100 mM EDTA). Cell lysate was separated via electrophoresis on an 8% SDS-polyacrylamide gel, followed by electrophoretic transfer to a Hybond-P PVDF membrane. The samples were and blocked with 5% skim milk in TBS with 0.1% Triton X-100 (TBS-T) for 120 min at room temperature. The membranes were then incubated overnight with anti-HSL and anti-phospho-HSL antibodies (1:1000 dilution, Cell signaling) in TBS-T at 4 °C. After incubation with horseradish peroxidase, anti-rabbit IgG antibody (1:2500, Cell signaling) was conjugated for 1 h at room temperature. For normalization, the membranes were reprobed with antibodies against β-actin (1:5000 dilution, Santa Cruz Biotechnology). Several exposure times were used to obtain the signals in the linear range.

### 3.5. Glucose Uptake Activity Assay

Glucose uptake activity was analyzed by measuring the uptake of 2-deoxy-d-[^3^H] glucose, as described previously. 3T3-L1 cells plated in a 24 well plate were washed three times with PBS and incubated in 1 mL/well warm PBS containing 10 μg/mL insulin for 30 min at 37 °C. After washing twice in PBS, the cells were incubated in 1mL low glucose DMEM containing cytochalasin B or fucoidan and 2-deoxy-d-[^3^H] glucose. After 30 min, glucose uptake was terminated by washing the cells three times with cold PBS. The cells were lysed with 0.1 mL of 1% SDS per well at RT for 10 min. The radioactivity retained by the cell lysates was determined using a scintillation counter (1450 MicroBeta TriLux, USA).

### 3.6. Triglyceride Accumulation Content Assay

3T3-L1 adipocytes, cultured with fucoidan at 200 μg/mL in each well of 6 well plates for 8 days, were harvested in 300 μL of 1% triton X-100 and then centrifuged. The triglyceride contents of the cell lysates were analyzed by using the TG-S reaction kit (Asan Pharm. Co., Seoul, Korea) according to the manufacturer’s instructions.

### 3.7. Statistical Analysis

The significant differences (* *P* < 0.05, ** *P* < 0.001) between samples were evaluated through the student’s *t*-test.

## 4. Conclusions

In this study, we showed fucoidan from marine brown algae inhibits lipid accumulation in 3T3-L1 adipocytes via stimulating lipolysis. The treatment of fucoidan reduced lipid accumulation in 3T3-L1 cells in a dose-dependent manner by determined Oil Red O staining, and a 52.2% decrease was observed at 200 μg/mL (*P* < 0.05). Fucoidan reduces the lipid accumulation by stimulating lipolysis from the enhancement of HSL and p-HSL expression. Therefore, these results suggest that fucoidan could be useful for prevention or treatment of obesity by its stimulatory lipolysis.

## References

[1] Larsson B, Svardsudd K, Welin L, Wilhelmsen L, Bjorntorp P, Tibblin G (1984). Abdominal adipose tissue distribution, obesity and risk of cardiovascular disease and death: 13 year follow up of participants in the study of men born in 1913. Br. Med. J.

[2] Spiegelman BM, Flier JS (2001). Obesity and the regulation of energy balance. Cell.

[3] Kopelman PG (2000). Obesity as a medical problem. Nature.

[4] Kawada T, Takahashi N, Fushiki T (2001). Biochemical and physiological characteristics of fat cell. J. Nutr. Sci. Vitaminol (Tokyo).

[5] Shi Y, Burn P (2004). Lipid metabolism enzymes: Emerging drug targets for the treatment of obesity. Nat. Rev. Drug. Discov.

[6] Chapman VJ, Chapman DJ (1980). Seaweeds and Their Uses.

[7] Patankar MS, Oehninger S, Barnett T, Williams RL, Clark GF (1993). A revised structure for fucoidan may explain some of its biological activities. J. Biol. Chem.

[8] Li B, Lu F, Wei X, Zhao R (2008). Fucoidan: Structure and bioactivity. Molecules.

[9] Kuznetsova TA, Besednova NN, Mamaev AN, Momot AP, Shevchenko NM, Zvyagintseva TN (2003). Anticoagulant activity of fucoidan from brown algae. Fucus evanescens of the Okhotsk Sea. Bull. Exp. Biol. Med.

[10] Maruyama H, Tamauchi H, Iizuka M, Nakano T (2006). The role of NK cells in antitumor activity of dietary fucoidan from Undaria pinnatifida sporophylls (Mekabu). Planta Med.

[11] Hayashi K, Nakano T, Hashimoto M, Kanekiyo K, Hayashi T (2008). Defensive effects of a fucoidan from brown alga Undaria pinnatifida against herpes simplex virus infection. Int. Immunopharmacol.

[12] Wang J, Zhang Q, Zhang Z, Li Z (2008). Antioxidant activity of sulfated polysaccharide fractions extracted from Laminaria japonica. Int. J. Biol. Macromol.

[13] Kim KJ, Lee OH, Lee BY (2010). Fucoidan, a sulfated polysaccharide, inhibits adipogenesis through the mitogen-activated protein kinase pathway in 3T3-L1 preadipocytes. Life Sci.

[14] Kim MJ, Chang UJ, Lee JS (2009). Inhibitory effects of fucoidan in 3T3-L1 adipocyte differentiation. Mar. Biotechnol.

[15] Rocha de Souza MC, Marques CT, Guerra Dore CM, Oliveira Rocha HA, Ferreira da Silva FR, Leite EL (2007). Antioxidant activities of sulfated polysaccharides from brown and red seaweeds. J. Appl. Phycol.

[16] Student AK, Hsu RY, Lane MD (1980). Induction of fatty acid synthetase synthesis in differentiating 3T3-L1 preadipocytes. J. Biol. Chem.

[17] Langin D, Holm C, Lafontan M (1996). Adipocyte hormone sensitive lipase: A major regulator of lipid metabolism. Proc. Nutr. Soc.

[18] Kim SO, Lee EJ, Choe WK (2006). The effects of Ginseng Saponin-Re, Rc and green tea catechine; ECGC (Epigallocatechin Gallate) on leptin, hormone sensitive lipase and resistin mRNA expressions in 3T3-L1 adipocytes. Korean J. Nutr.

[19] Cha SY, Jang JY, Lee YH, Lee G, Lee HJ, Hwang KT, Kim YJ, Jun WJ, Lee JM (2010). Lipolytic effect of methanol extracts from *Luffa cylindrica* in mature 3T3-L1 adipocytes. J. Korean Soc. Food Sci. Nutr.

[20] Newsholme EA (1976). Carbohydrate metabolism *in vivo*: Regulation of the blood glucose level. Clin. Endocrinol. Metab.

